# Association between social engagement frequency and depression among the older people in China: evidence from the 2011–2018 China Health and Retirement Longitudinal Study

**DOI:** 10.1136/bmjph-2023-000601

**Published:** 2024-09-18

**Authors:** Jining Li, Xingna Zhang, Benjamin Barr

**Affiliations:** 1 Department of Health and Environmental Sciences, Xi'an Jiaotong-Liverpool University, Suzhou, Jiangsu, China; 2 Department of Public Health, Policy and Systems, University of Liverpool, Liverpool, UK

**Keywords:** Public Health, Education, Age Factors, Mental Health

## Abstract

**Background:**

Depression in older people is a major concern in China. Despite being known as a protective factor, it is unclear the extent to which social engagement mitigates depression or mediates socioeconomic mental health inequalities among Chinese older people.

**Methods:**

We conducted a survival analysis on 2891 respondents aged 55–77 without depression in 2011 from the Chinese Health and Retirement Longitudinal Study to estimate the relative risk of developing depression between 2011 and 2018 with different social engagement frequencies, having adjusted for age, sex, education, marriage, employment, household income, self-reported physical health and urban/rural residency. We then assessed the potential mediation effect of social engagement on the association between social class (measured by education) and depression.

**Results:**

Respondents who engaged in social activities almost daily were 15% less likely to develop depression (HR 0.85, 95% CI 0.74 to 0.98) than those who never. Respondents with secondary education and above were 20% less likely to develop depression (HR 0.80, 95% CI 0.69 to 0.94) and 60% more likely to engage in social activities (HR 1.60, 95% CI 1.48 to 1.74) than those with elementary education and below. After controlling social engagement frequencies, the effect of education on depression changed very little (HR 0.82, 95% CI 0.70 to 0.96).

**Conclusion:**

As an important modifiable protective factor, we however found no clear evidence of social engagement mediating between social class and depression. Future social policies should aim to improve well-being by promoting social engagement to prevent depression among Chinese older people.

**WHAT IS ALREADY KNOWN ON THIS TOPIC**

- Social engagement is associated with a reduced risk of depression, but limited evidence on its causal effect on preventing/reducing depression among older people in China. Not to mention its potential mediating role between social class and depression.

**WHAT THIS STUDY ADDS**

- This study examined the preventative role of social engagement on depression among older people in China, within a possible causal framework using a longitudinal study design.
- Social engagement did not appear to be a mediator between social class and depression among older people in China.

**HOW THIS STUDY MIGHT AFFECT RESEARCH, PRACTICE OR POLICY**

- Policies that promote social engagement among older people in China are likely to reduce risk of depression, further work is needed to understand how to promote social activities that reduce inequalities in depression.

## Introduction

 China is ageing rapidly, with the largest older population (people aged ≥65 years amounted to 200.6 million, 14.2% of the total population in 2022) in the world currently.^1^ Many reasons account for this while the continued socioeconomic growth and improved access to healthcare since the 1990s have boosted life expectancy,^2^ the one-child policy restricting most families to have one child only within each household between the 1980s and 2016 has created a generation of much fewer younger people drastically below the natural fertility rate. Ageing in China has become a demographic challenge that has significant social, economic and policy implications including impacts on labour markets, the pension system, the health and social care system and the traditional family-based elderly care system.^3^ The rapid economic development, growing individualistic values and shrinking family sizes have led to a decline in the social status of older people in China, along with a rapid increase in the number of the older population.^4^ It is important for policy-makers to develop effective interventions for healthy ageing to address the challenges brought by this demographic shift.

Among the numerous challenges of the ageing population in China, senior citizen’s mental health is a major concern.^5 6^ Compared with physical health, mental health still receives insufficient attention and inadequate resource allocation due to the associated stigma.^5^ Depression in later life, in particular, has a 20% overall prevalence^7^ with much negative health outcomes^8^ including increased morbidity^5^ and mortality,^9^ reduced quality of life,^10^ and increased burden to the healthcare system. A meta-analysis investigated the prevalence of depressive symptoms among older people (≥60 years old) in China and found that the pooled prevalence of depressive symptoms was 22.7% (95% CI 19.4% to 26.4%) and that it decreased with increasing levels of education.^11^ As a major public health problem, it is, therefore, crucial to investigate the risk and protective factors of depression in older people. Major risk factors include chronic diseases and disability/impairment while physical activity shows protective effects.^12^ In one study in China depression was significantly negatively associated with ageing, lower household income, deterioration of physical conditions and lower social support.^13^ Yan *et al*^14^ found that compared with married older people, unmarried older people (including the widowed, divorced and never-married) had a higher risk for depression. Bjelland *et al*^15^ found low educational levels were significantly associated with depression.

A focus on social engagement is highly relevant in China due to its modifiable nature. The WHO has recognised mental health promotion as a global priority, emphasising the importance of modifiable social determinants of health.^16 17^ Among all the socioeconomic and cultural factors related to mental health, social engagement is one of the most suitable and effective factors to promote at the population level. Engaging in social activities does not require specialised training or intervention, and many community-based initiatives can be easily implemented, modified and are low-cost. Changing one’s social engagement can be relatively easy, inexpensive and quite effective to promote mental health among older people.^18 19^ However, Chinese policy-makers inadequately use social engagement in designing targeted intervention measures, as evidenced by the absence of social engagement interventions in the first action plan for the prevention and treatment of depression released by the National Health Commission of China in 2020—‘The Action Plan to Develop Specialized Services for the Prevention and Treatment of Depressive Disorders’.^20^ While the Action Plan outlines a range of strategies for prevention and treatment of depression, it does not include the detailed prevention measures for the older population while overlooking the broader social determinants. This study suggests a need to expand the focus to consider social engagement in addressing depression. Existing literature suggests that social engagement has a strong association with depression in older people.^21 22^ Social engagement, defined as leisure or productive activities that are related to physical and psychological health,^21 23^ is an important protective factor for depression in older people.^21^ Engaging in social activities can enhance one’s social support and social belongingness,^24^ which can further help to reduce or mitigate depressive symptoms among older people.^25^ Engaging in social activities could also maintain and enhance well-being^26^ and is an indicator of healthy ageing.^27^ Frequent social engagement was associated with longevity,^28^ and continuously or initiating social engagement reduces depressive symptoms among older people.^25^ Previous research in China using the Chinese Health and Retirement Longitudinal Study (CHARLS) datasets found that social engagement improved the self-rated physical health and reduced mental distress but had no effect on chronic disease status,^29^ and that attending social activities weekly or more often reduced the risk of depressive symptoms.^30^

However, socioeconomic inequalities present in social engagement and depression in older people. Health inequalities commonly refer to the distribution of health by socioeconomic position in the UK.^31^ While variations in health occur naturally, social inequalities in health are systematic, socially produced, unfair, unjust and unnecessary.^32 33^ Socioeconomic inequalities in health occur when there are systemic health differences between groups with unequal social statuses or classes.^34^ Link and Phelan^35^ argued that socioeconomic status is a fundamental cause of health inequalities, which is linked with resources such as money, knowledge, power, prestige and various social connections. They also highlighted the more advantaged members of society, that is, the individuals with higher socioeconomic status, they had higher access to flexible resources and that gave them greater adaptability. The privileged position of these individuals affords them easier access to crucial resources to protect themselves from health risks, for example, accessing new health information, adopting health technologies and changing behaviour in response to health risks.^35^ Social class is commonly measured by income, occupation and/or education. Education is widely used in measuring social class in public health research.^36^ In our study, education was chosen as the measure of social class for its ease of measurement, its relevance to individuals outside of the active workforce, its relative stability throughout one’s lifespan regardless of changes in health status, and its association with a multitude of health outcomes.^36^,^38^ While income is also a critical aspect of social class, it was used as a confounder in this study. First, this study employed the metric of annual household income, which potentially dilutes the impact of individual financial status. Second, this study mainly focused on the older population, they might have reduced income due to retirement or decreased earning capacity due to health conditions.

Arpino and Solé-Auró found that a higher educational level was associated with better self-perceived health and fewer depressive symptoms among European older people.^39^ The ability to engage socially is often influenced by one’s socioeconomic status. Arpino and Solé-Auró focused on engagement in three types of active ageing activities and examined it as mediators of the effect of education on health outcomes: (1) social engagement, (2) paid work and (3) grandchild care. Differences in levels of engagement in active ageing explained up to a third of the health disparities between higher and lower-educated groups. Policies encouraging active social engagement among older individuals should specifically target those with lower education levels, so as to reduce health inequalities linked to educational background and benefit overall well-being and mental health, including depression.^39^

Although an extensive body of literature has explored the relationship between social engagement and depression in older people,^21 22 25 30^ there are several important gaps in the current evidence base. First, previous cross-sectional studies have conducted limited investigation on the causal effect of social engagement on depression among Chinese older people, due to the inherent limitation of cross-sectional data in establishing temporal precedence.^40^ A causal perspective can enhance our understanding by deriving models to test crucial hypothesised causal pathways, for example, using survival models to discover the causal effect of social engagement on new onsets of depression as in our study. Second, little is known about the extent to which social engagement acts as a mediator in the effect of social class on depression among the older population. Understanding this mediating mechanism is crucial because it could offer insights into how social engagement mitigates or exacerbates the mental health disparities observed across different social classes. This study seeks to examine this understudied mediating mechanism to provide additional evidence on comprehensively addressing problems of health inequalities.

We therefore endeavour to bridge both research gaps, by revealing the protective potential of social engagement on depression and examining the mediating mechanism of social engagement frequency between social class and depression among Chinese older people. The research questions are ‘does social engagement reduce the risk of new-onset depressive symptoms in later life among older people in China?’ and ‘is social engagement a mediator in the effect of social class on depressive symptoms among Chinese older people?’. We highlight the hypothesised causal pathways for each question in directed acyclic graphs in online supplemental section A and use these to inform our analysis.

## Methods

### Data and study population

In this study, we used harmonised CHARLS data from waves 1 to 4 (2011–2018),^41^ containing 25 586 respondents in total. CHARLS was conducted by Peking University to collect a nationally representative sample of Chinese residents aged 45 and older for studying the ageing population. The baseline survey (wave 1) of CHARLS was done in 2011. The respondents were then followed up every 2 or 3 years until 2018, with a wide range of sociodemographic information collected. We selected respondents aged between 55 and 69 and not depressed at wave 1 (2011), excluding those with missing data or having not participated in all four waves of measurements. The selection criteria are necessary for survival analysis to examine the respondents without depression at the baseline and the probability of developing depression by ageing. The decision on the age range was primarily informed by the retirement age in China, which is 55 for women and 60 for men. By selecting this age range, we aimed to capture a transitional period in the lives of our respondents, encompassing both the preretirement and postretirement phases. By the time of wave 4, these respondents were aged between 55 and 77. This extended age range ensured that our study not only covers the critical retirement transition but also includes a period where individuals are typically still active and engaged in social activities (see online supplemental section B for employment and unemployment rate in 2011).

**Figure 1:**
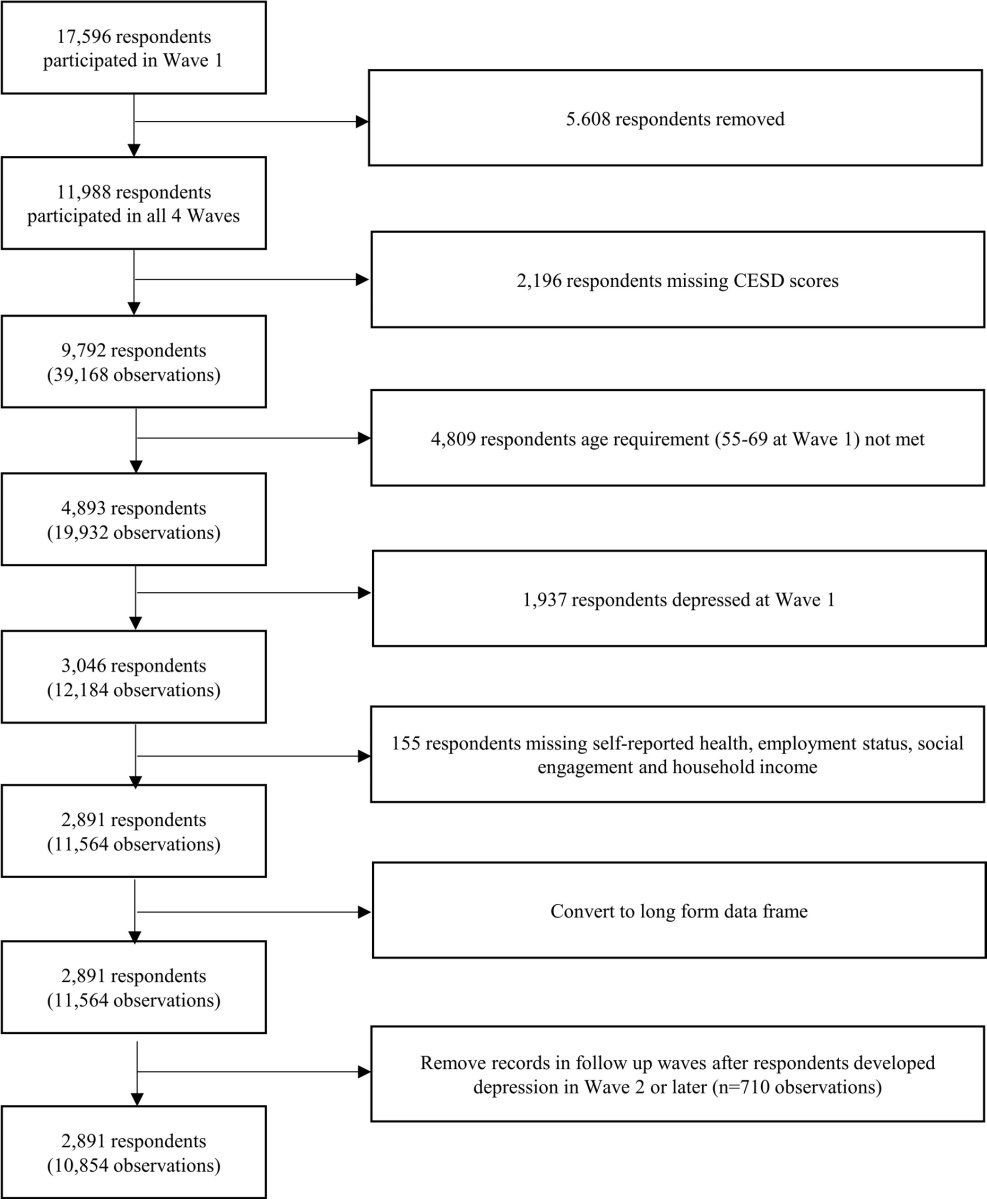
Participants’ inclusion flow in the study. CESD, Centre for Epidemiological Studies–Depression.

Figure 1 is the flow chart showing how we constructed the sample. We started with a total of 17 596 respondents contained in the CHARLS wave 1, among whom 11 988 respondents had participated in all 4 waves. We then excluded 2196 respondents with missing Centre for Epidemiological Studies–Depression Scale (CESD) scores for any of the four waves (including death, non-enrolment and non-response), 4809 respondents with age outside the 55–69 range at wave 1, 1937 respondents with depressive symptoms at wave 1, and 155 respondents with missing data on any of the key confounders. After obtaining the sample of 2891 respondents who did not have depression in wave 1 and who had non-missing data on all confounders across all 4 waves, we had 11 564 observations in total (n=2891×4). In order to construct a discrete-time hazard model of the onset of new depression, it was crucial to remove observations post the onset of depression. Therefore, for any respondent who developed depression in wave 2 or later, their subsequent observations were excluded from the analysis as they no longer met the criteria of being ‘depression-free survivors’. Finally, we reached the sample of 10 854 observations.

### Measurement

#### Measuring depression

Depressive symptoms were measured by the 10-item CESD score in CHARLS. The 10-item questionnaire measured how participants felt and behaved during the past week. The 10 items included ‘I was bothered by things that don’t usually bother me’, ‘I had trouble keeping my mind on what I was doing’, ‘I felt depressed’, ‘I felt everything I did was an effort’, ‘I felt hopeful about the future’, ‘I felt fearful’, ‘My sleep was restless’, ‘I was happy’, ‘I felt lonely’ and ‘I could not get going’. Each answer was assigned with a value ranging from 0 to 3. The total score was then calculated by summing up the values of the 10 items. A score of 10 and above was considered depressed in this study, following the routine practice in the existing literature.^42 43^ Our outcome variable was defined as a binary categorical measurement with 1 representing depression (a score of 10 and above) and 0 otherwise.

#### Measuring social engagement frequency

Our main exposure variable was social engagement frequency, a categorical variable measuring the frequency of respondents’ social engagement in the past month. In the CHARLS questionnaire, the respondents were asked whether they had done a wide range of social engagement activities in the last month: interact with friends; played Ma-jong, played chess, played card or went to community club; provided help to family, friends or neighbours who do not live with you and who did not pay you for the help; went to a sports, social or other kind of club; took park in a community-related organisations; done voluntary or charity work; cared for a sick or disabled adult who does not live with you and who did not pay you for the help; attended an educational or training course; stock investment; used the internet; other and none of these. The respondents were then asked: How often in the last month have you done these activities? Almost daily, almost every week or not regularly? In this study, a value of 1 represented that the respondent never participated in any social activities, 2 stood for irregular participation, 3 denoted weekly participation and 4 meant almost daily participation.

#### Measuring social class

We used educational status to measure social class, which was a binary categorical variable in our study with 1 representing secondary education and above and 0 representing elementary education and below. The consolidation into two categories was driven by a need to enhance the statistical power by focusing on broader distinctions in educational attainment that are likely to have more pronounced impacts on the outcomes of interest. The recoding aligns with the research objectives, which focus on examining significant educational divides (social class) that might influence depression. The binary division allows for clearer comparisons and interpretations within the context of these objectives. Furthermore, the educational distribution at wave 1 showed that respondents with an educational status of elementary or below constituted 71.5% of the sample, respondents with an educational status of secondary education and above constituted 28.5% of the sample. Recoding it as ≤elementary and ≥secondary could add statistical power to this analysis.

#### Social, economic and demographic factors

In order to estimate the causal effect of social engagement on depression, we adjusted for confounders identified as having associations with both social engagement and depression in this study. These included age, sex, educational status, annual household income, residential area, self-reported physical health status, marital and employment status. After deflating annual household income with the respective rural and urban price indexes between 2011 and 2018 (the baseline year for price indexes in this study) from the 2019 Chinese Statistical Yearbook,^44^ we imputed missing values of household income with the mean across the four waves for each individual, respectively. We then recategorised the annual household income as a binary variable based on its median (CN¥24 810), with 1 representing the median and above and 0 otherwise. Residential area was a binary variable as well with 0 representing living in urban areas and 1 for rural areas. Self-reported physical health status served as a subjective reflection of respondents’ perceived physical health and it was categorised into three categories: very good and good, fair and poor and very poor. Marital status was binary with 0 representing being married and 1 otherwise. Employment status was classified as working (employed or self-employed) and non-working (retired or unemployed) being represented by 0 and 1 respectively.

### Statistical analysis

We employed survival analysis because we are interested in understanding the risks and protective factors influencing the new onset of depression among people who are reaching retirement age in good mental health. This is of particular public health relevance as there is the potential to reduce this burden by preventative policy interventions targeting people in retirement. The persistence of pre-existing depression into retirement may be less amenable to intervention. Looking at the relationship between baseline risk factors and new onset within a survival framework, we believe also helps disentangle the causal direction of effect, compared with, for example, modelling recurrence and recovery of depression over time, where there are potential bidirectional effects between social participation and depression. Using survival analysis in longitudinal data allows us to follow respondents who were free of depression at baseline and investigate whether social engagement frequency is associated with the onset of new depression over the period. To visualise the onset of depression and how this differed by level of social engagement we initially plotted survival curves showing the proportion of our sample who remained free of depression as they aged. These survival curves were plotted separately for two exposure groups (individuals who never engaged in social activities and those who engaged almost daily) and for each of the confounders. To investigate the independent effect of social engagement on depression onset, we fitted a discrete-time survival model, additionally controlling for the confounders. We used a binomial generalised linear model with log-log link function, treating age as the time-to-event variable. Fitting such a model with grouped survival data provided an analogue of the continuous proportional hazards model^45^ to investigate the relationship between these survival times and a set of explanatory variables.

To investigate whether social engagement mediated the effect of social class on depression onset we used the three steps in Baron and Kenny’s mediation model.^46^ First, we estimated a discrete-time hazard model, as outlined above, to investigate how educational status predicted depression, controlling for potential confounders of this relationship (sex, marital status, self-reported physical health status and residential area), second we estimated a regression model investigate how educational status predicted social engagement frequency, controlling for potential confounders of this relationship (sex, marital status, self-reported physical health status and residential area). As the social engagement outcome was ordinal, we used an ordinal logistic regression rather than a complementary log-log model. Third, we updated the first regression model including both educational status and social engagement frequency (the mediator in this analysis) to investigate how educational status predicted depression, while controlling for social engagement. In this analysis, we determine that social engagement mediates the relationship between education and depression if, education is a predictor of depression in the first model and education is a predictor of social engagement in the second model and the effect of education on depression is greatly reduced in the third model when controlling for social engagement. We conducted all the analyses using R V.4.2.2.

We performed four different sensitivity tests to check if the following variations would impact the findings: (1) bivariate survival analyses by further restricting all respondents’ age to be below 74 in online supplemental section C.1; (2) bivariate survival analyses by sex in online supplemental section C.2; (3) including quadratic and cubic terms of age in the general linear model in online supplemental section C.5 and (4) using age as a continuous variable in online supplemental section C.4. In online supplemental section D, we investigated the effect of social engagement on depression among Chinese older people.

### Patient and public involvement

In the course of conducting this research, we did not directly involve patients or the public. This study primarily focused on secondary data analysis using the dataset of CHARLS and we did not have contact or identification information about the respondents, and as such, patient and public involvement (PPI) was not incorporated into the research process.

## Results

### Descriptive statistics

Table 1 shows the summary statistics of all the variables used in this study by each wave, with a total sample of 2891 respondents who participated in all 4 waves, aged 55–69 and not depressed at wave 1 (see online supplemental section E for baseline descriptive statistics). The observed decrease in the percentage of respondents with CESD scores of 10 or above in later waves can be attributed to our methodological design, specifically the exclusion of observations from the analysis once the respondents develop depression in wave 2 or later. This approach allows for a focused examination of depression onset and survival, rather than changes in existing depressive symptoms.

**Table 1:** Summary of the social, economic and demographic characteristics of the sample

Respondents=2891	Wave1 (percentage)		Wave2 (percentage)		Wave3 (percentage)		Wave4 (percentage)	
Observations=10 854	2891		2891		2627		2445	
Age groups								
55–59	1309	(45.3)	812	(28.1)	218	(8.3)	0	(0.0)
60–69	1582	(54.7)	1861	(64.4)	1982	(75.4)	1650	(67.5)
70–74	0	(0.0)	218	(7.5)	427	(16.3)	618	(25.3)
≥75	0	(0.0)	0	(0.0)	0	(0.0)	177	(7.2)
Sex								
Men	1581	(54.7)	1581	(54.7)	1488	(56.6)	1398	(57.2)
Women	1310	(45.3)	1310	(45.3)	1139	(43.4)	1047	(42.8)
Marital status								
Married	2554	(88.3)	2516	(87.0)	2260	(86.0)	2028	(82.9)
Not married	337	(11.7)	375	(13.0)	367	(14.0)	417	(17.1)
Educational status								
≤Elementary	2068	(71.5)	2068	(71.5)	1856	(70.7)	1700	(69.5)
≥Secondary	823	(28.5)	823	(28.5)	771	(29.3)	745	(30.5)
Residential area								
Urban	1065	(36.8)	1065	(36.8)	988	(37.6)	944	(38.6)
Rural	1826	(63.2)	1826	(63.2)	1639	(62.4)	1501	(61.4)
Self-reported physical health status								
Good and very good	822	(28.4)	768	(26.6)	719	(27.4)	646	(26.4)
Fair	1550	(53.6)	1649	(57.0)	1525	(58.1)	1306	(53.4)
Poor and very poor	519	(18.0)	474	(16.4)	383	(14.6)	493	(20.2)
Total household income								
Below the median	1446	(50.0)	1446	(50.0)	1288	(49.0)	1180	(48.3)
Above the median	1445	(50.0)	1445	(50.0)	1339	(51.0)	1265	(51.7)
Employment status								
Working	1959	(67.8)	1954	(67.6)	1656	(63.0)	1439	(58.9)
Non-working	932	(32.2)	937	(32.4)	971	(37.0)	1006	(41.1)
Frequency of attending social activities								
Never	1427	(49.4)	1427	(49.4)	1284	(48.9)	1187	(48.5)
Not regularly	398	(13.8)	398	(13.8)	366	(13.9)	343	(14.0)
Almost every week	325	(11.2)	325	(11.2)	297	(11.3)	269	(11.0)
Almost-daily	741	(25.6)	741	(25.6)	680	(25.9)	646	(26.4)
CESD score								
0~9	2891	(100.0)	2317	(80.1)	2239	(85.2)	2106	(86.1)
10~30	0	(0.0)	574	(19.9)	388	(14.8)	339	(13.9)

CESDCentre for Epidemiological Studies–Depression

### Survival analysis

**Figure 2:**
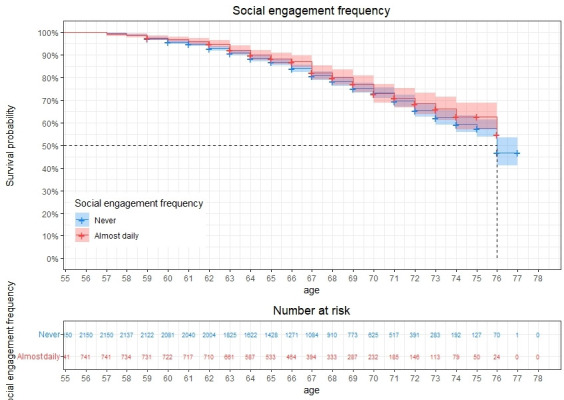
Survival curve of social engagement frequency in developing depression for age 55–77, with 95% CIs.

Figure 2 shows the survival curve (percentage remaining free of depression) for two groups of respondents based on their level of social engagement at baseline, comparing the survival probability of individuals who never engaged in social activities and those who engaged in social activities almost daily. Over time the percentage who remain free of depression in the socially engaged group remains slightly higher than in the group that never engaged in social activities. This suggests that engaging in social activities more frequently is associated with stronger protective effects against depression, particularly among older individuals.

We found that six out of seven confounders were strongly associated with the onset of depression between the ages 55 and 77 in univariate analysis. Probability of depression was higher among women, and people with lower education, from poorer households, living in the rural areas, not working and with poorer self-reported physical health. No difference was found for groups of different marital statuses (see online supplemental section F).

### Effects of social engagement frequency on depression

Table 2 shows the association between social engagement frequency (measured in wave 1) and depression among Chinese older people after adjusting for the confounders (sex, educational status, annual household income, residential area, self-reported physical health status, marital status and employment status). We found that only the ‘almost daily’ group showed a significant difference from the reference group and were 15% (HR 0.85, 95% CI 0.74 to 0.98) less likely to develop depression.

**Table 2:** Estimates of the effect of social engagement frequency on developing depression among respondents aged 55–77, controlling age, sex, educational status, annual household income, residential area, self-reported physical health status, marital status and employment status

		HR	95% CI	P value
Frequency of social activities (Reference: never)				
	Not regularly	0.950	(0.806, 1.121)	0.544
	Almost every week	0.951	(0.790, 1.144)	0.592
	Almost daily	0.847	(0.736, 0.975)	0.021
Age		1.032	(1.020, 1.044)	<0.001
Sex (Reference: men)				
	Women	1.408	(1.256, 1.579)	<0.001
Educational status (Reference: ≤elementary)				
	≥Secondary	0.866	(0.750, 1.000)	0.049
Marital status (Reference: married)				
	Not married	1.316	(1.140, 1.518)	<0.001
Self-reported physical health status (Reference: good and very good)				
	Fair	1.787	(1.522, 2.097)	<0.001
	Poor and very poor	3.550	(2.985, 4.221)	<0.001
Residential area (Reference: urban)				
	Rural	1.230	(1.078, 1.403)	0.002
Employment status (Reference: working)				
	Non-working	0.905	(0.798, 1.206)	0.120
Household income (Reference: below the median)				
	Above the median	0.837	(0.744, 0.942)	0.003

### Mediation analysis

The focus in this analysis is on the relationship between educational status, depression and whether this is mediated by social engagement frequency. Table 3 and online supplemental section G show each of the models in Barron and Kenny’s steps for mediation analyses. Model 1 shows that higher educational status (secondary education and above) is clearly associated with reduced risk of depression (HR 0.80, 95% CI 0.69 to 0.94), when adjusting for confounders (sex, marital status, self-reported physical health status and residential area). Model 2 (see online supplemental section G for full model output) shows that respondents with secondary education and above were around 60% more likely to engage in social activities more frequently than those with elementary education and below (HR 1.60, 95% CI 1.48 to 1.74). However, model 2 in table 3 shows the association between educational status (as our exposure) and depression, when controlling for social engagement as a mediator (while also controlling for other confounders). The HR for respondents with secondary education and above of model 2 remains very similar (HR 0.82, 95% CI 0.70 to 0.96) when compared with model 1 (HR 0.80, 95% CI 0.69 to 0.94). By comparing these two models, we conclude there is no strong evidence to support the mediation effect.

**Table 3:** Association between educational status and depression when controlling social engagement as a mediator among respondents aged 55–77

		Model 1			Model 2		
		HR	95% CI	P value	HR	95% CI	P value
Educational status (Reference: ≤elementary)							
	≥Secondary	0.807	(0.694, 0.937)	0.005	0.821	(0.706, 0.955)	0.011
Frequency of social activities (Reference: never)							
	Not regularly				0.943	(0.788, 1.127)	0.517
	Almost every week				0.925	(0.757, 1.130)	0.446
	Almost daily				0.806	(0.693, 0.937)	0.005
Age		1.034	(1.022, 1.047)	<0.001	1.034	(1.022, 1.047)	<0.001
Sex (Reference: men)							
	Women	1.414	(1.251, 1.598)	<0.001	1.429	(1.264, 1.616)	<0.001
Marital status (Reference: married)							
	Not married	1.350	(1.153, 1.579)	<0.001	1.355	(1.158, 1.586)	<0.001
Self-reported physical health status (Reference: good and very good)							
	Fair	1.836	(1.553, 2.171)	<0.001	1.834	(1.551, 2.169)	<0.001
	Poor and very poor	3.923	(3.264, 4.716)	<0.001	3.903	(3.247, 4.692)	<0.001
Residential area (Reference: urban)							
	Rural	1.391	(1.217, 1.588)	<0.001	1.363	(1.193, 1.558)	<0.001

## Discussion

Our study shows that daily social engagement is associated with a decreased risk of depression among older people in China, this study contributes to the existing literature by revealing the possible causal mechanisms. To the best of our knowledge, this study is the first to explore the potential causal relationship between social engagement and depression in older people using the longitudinal dataset of CHARLS from 2011 to 2018. Results of this study confirm that social engagement is an important protective factor for the development of depressive symptoms among the respondents. This study is also the first to explore whether social engagement mediates the relationship between social class and depression among the respondents. We did not observe strong evidence for mediation within our study, suggesting that there might be other factors mediating the relationship between social class and depression. For instance, material circumstances such as income, social status or housing conditions could be contributors to the unequal differences in depression among different educational statuses.

### Strengths and limitations

Our study has a number of strengths. By using a nationally representative longitudinal dataset (CHARLS), the findings of this study have a good potential for generalisability and transferability. Our study has provided evidence supporting a possible causal relationship between social engagement and depression using a longitudinal design with robust confounder adjustment. Our study also accounts for the potential influence of reverse causation that depression may reduce social engagement, by controlling respondents to be without depression at the start of the study.

Our study has limitations. First, we conducted secondary analysis of CHARLS data, where limited information on social engagement frequency in the past month was captured. Future studies can collect primary and more detailed data on social engagement frequency for a longer period and different types of social engagement. As different sexes may have differentiated preferences and frequencies for different types of social engagement, the availability of such data could further facilitate investigating the relationship between sex, social engagement type/frequency and depression among older people. Second, the physical health status was subjectively self-reported by respondents without a uniform standard, which may be inaccurate. Third, our study used the CESD-10 for measuring depression, as sourced from the CHARLS dataset, which relied on self-reports from the respondents. Fourth, as our analysis included only respondents who were alive across all four waves, there is a potential limitation of survivor bias. The observed decrease in depressive symptoms over time may be influenced because respondents with higher initial symptoms could have had a higher mortality rate. Lastly, we took values of social engagement frequency at wave 1 and treated it as time-fixed across four waves in our study. While such a treatment was necessary and important for testing the causal link between social engagement frequency and depression onset, we endeavour to examine the respective time-varying measures and their implications in our future research. The fact that social engagement frequency was treated as time-fixed across the four waves in the study could potentially lead to an underestimation of the mediating effect of social engagement on the relationship between social class and depression among older people in China. By not accounting for potential changes in social engagement over time, the study may not have captured the full extent of how social engagement influences the development of depression.

### Policy implications

Our findings suggest that social engagement has important potential as policy interventions to maintain and enhance psychological well-being among Chinese older people. The government should implement measures aimed at encouraging senior citizens to participate in social activities, for example, the establishment of community clubs for older people, promoting diverse activities and investing in counselling services tailored for older people. The finding of no mediation of social engagement on the relationship between social class and depression among older people in China suggests that social engagement does not fully account for the significant disparities in depression rates among different social classes. Interventions and policies should not only focus on individual-level factors but also address broader structural and institutional causes of mental health disparities. By tackling underlying social determinants of depression like poverty, housing, rural–urban inequalities and employment, a more comprehensive approach can be adopted to enhance mental well-being among older people and reduce the unequal burden of depression across various social classes in China.

## Conclusions

This research provides valuable insights to examine the possible causal relationship between social engagement and depression among Chinese older people. This research also addresses gaps within the literature by exploring the mediating mechanisms of social engagement on the relationship between social class and depression among older people in China. Social engagement is identified as an important protective factor against depression among older people in China and potentially in other societies with similar contexts. However, it is important to note that promoting social engagement only may not effectively address the inequalities in depression among the older people. To address health inequalities in depression, policies should therefore encompass a holistic approach, addressing these broader socioeconomic determinants to effectively enhance the quality of life and social engagement of the older population. For instance, initiatives to provide financial support for depression treatments and ensure economic security for vulnerable populations are crucial. In addition, targeted interventions in low socioeconomic communities such as community-based support programmes can also help to bridge the gap in mental healthcare accessibility. Our study also lends support to policy-makers to make informed decisions on designing social engagement interventions to promote healthy ageing for the older population.

## supplementary material

10.1136/bmjph-2023-000601 online supplemental file 1

## Data Availability

Data are available in a public, open access repository.
