# Supplementary material for: Association between social engagement frequency and depression among the older people in China: evidence from the 2011–2018 China Health and Retirement Longitudinal Study
Source: BMJ Public Health. 2024 Sep 18;2(2):e000601. doi: 10.1136/bmjph-2023-000601 (PMC11816298; doi:10.1136/bmjph-2023-000601)
Supplement: online supplemental file 1 [file bmjph-2-2-s001.pdf]

Supplementary Materials

Association between social engagement frequency and depression among the older people in China: Evidence from the 2011-2018 China Health and Retirement Longitudinal Study

Supplementary Section A. Directed Acyclic Graphs

Figure 3 below shows our directed acyclic graph (DAG) for our first research question. In the first research question, education is treated as a potential confounding variable. This means that education is included in the analysis as a confounder to control its potential influence on the relationship between social engagement frequency and depression. By estimating the association between social engagement frequency and onset of depression whilst controlling for sex, educational status, annual household income, residential area, self-reported physical health status, marital status and employment status, the aim is to isolate the direct effect of social engagement frequency on depression.

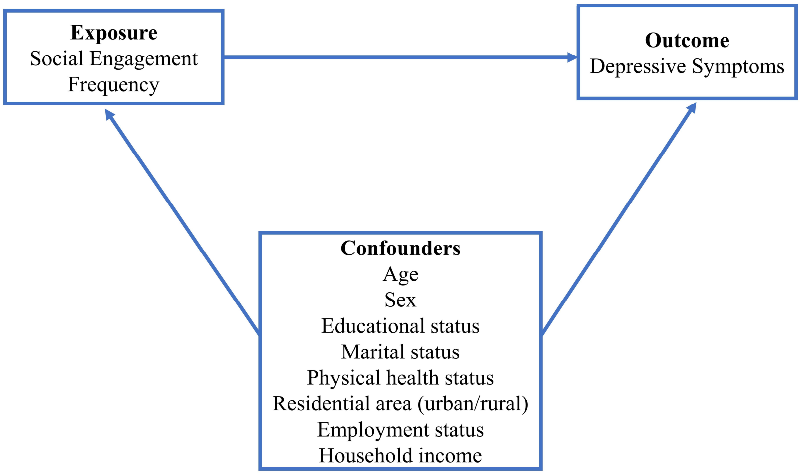

Figure. 3. A Directed Acyclic Graph (DAG) showing the hypothesized causal pathway running from social engagement frequency to depression.

Figure 4 gives the DAG for our second research question, here education is the exposure variable that measures social class, the outcome is depression, and social engagement frequency is a mediator. The aim is to examine whether the effect of education on depression is explained by the mediating role of social engagement frequency. We additionally control for sex, marital status, self-reported physical health status and residential area as confounders in this analysis. We do not include employment status and annual household income in this mediation analysis as they are also likely to be mediators in the relationship between education and depression, as for example education will influence employment status that will influence depression risk. In other words, they will be on the direct pathway between education (exposure) and depression (outcome) and will not be confounders (common causes of both education and depression).

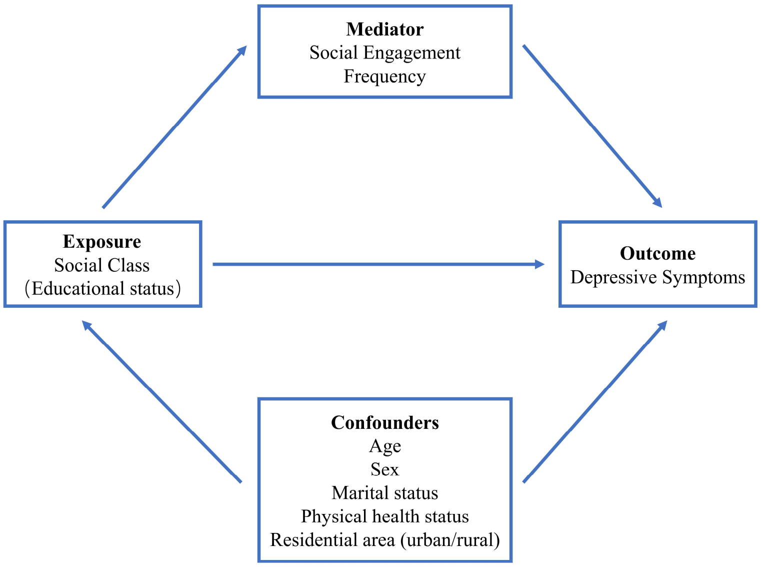

Figure. 4. A Directed Acyclic Graph (DAG) showing a hypothesized causal pathway running from social class to depression with social engagement frequency act as mediator.

Supplementary Section B. Employment distribution in older workers

The retirement age in China is typically 55 for women and 60 for men. The data source of figure 5 and 6 is Survey and Research Center for China Household Finance, each showing the employment and unemployment rate in 2011 respectively.

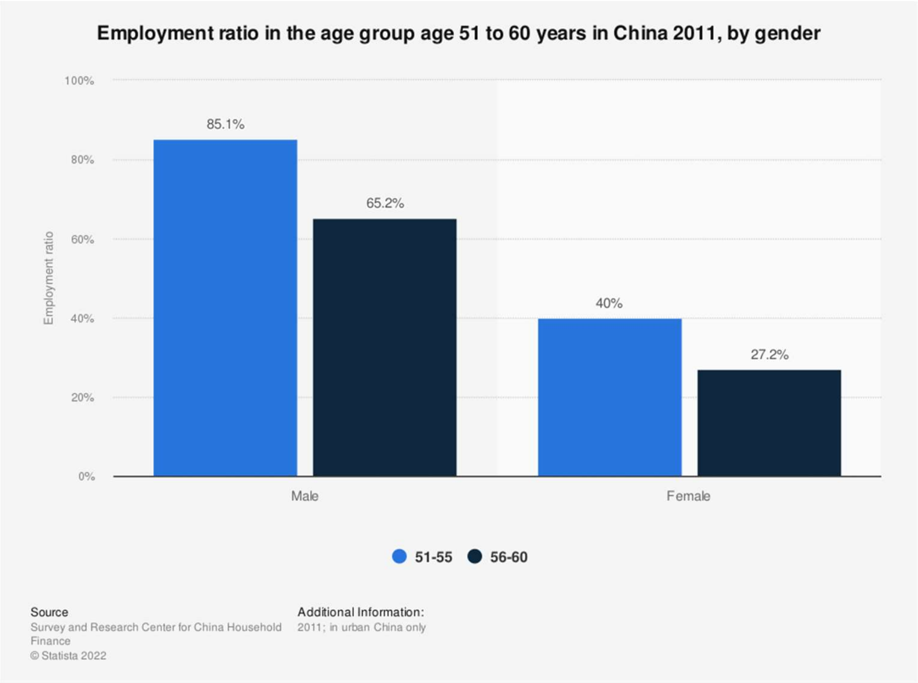

Figure. 5. Employment rate in age groups of 51-55 and 56-60 by sex in China in 2011 (Dara source: Survey and Research Center for China Household Finance).

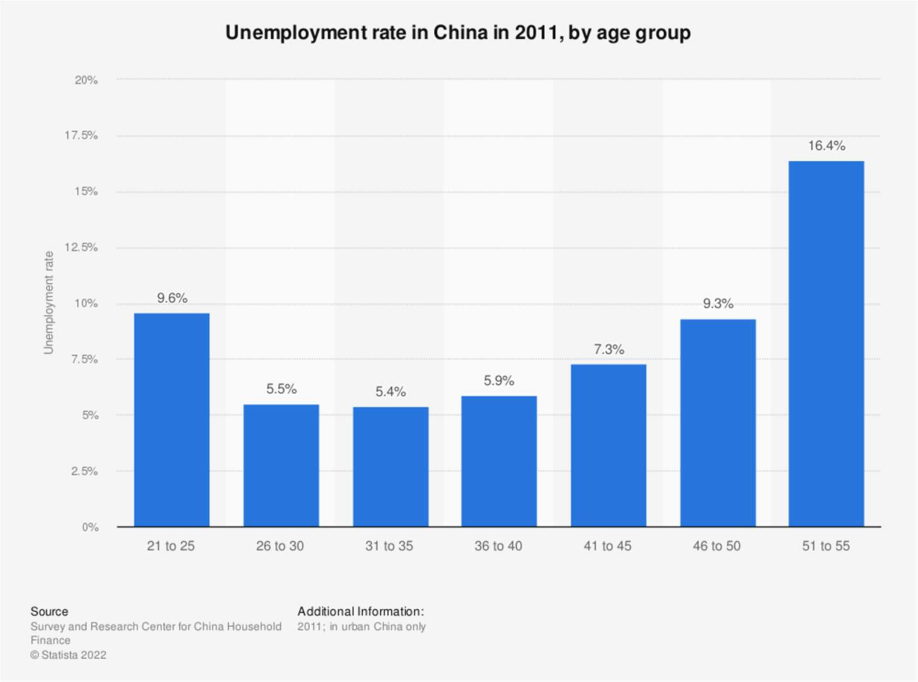

Figure. 6. Unemployment rate in China in 2011, by age group (Dara source: Survey and Research Center for China Household Finance).

Figure 5 shows that in 2011, 27.2% women aged 56 to 60 years were employed in urban China, while the employment rate for men is 65.2%.(47) By contrast, Figure 6 shows that 16.4% people (both women and men) aged 51-55 in urban China were registered as unemployed. (48)

Supplementary Section C. Sensitivity analyses

We performed four different sensitivity tests to check if the following variations would impact the findings: 1) bivariate survival analyses by further restricting all respondents' age to be below 74; 2) bivariate survival analyses by sex; 3) including quadratic and cubic terms of age in the general linear model; and 4) Using age as continuous variable.

Supplementary C.1 Bivariate survival analysis with respondents aged above 74

In the main analysis we had a sample of 2891, with a total of 10854 observations. The observations with age above 74 were deleted for sensitivity analysis because the percentage was too small for risk rate estimation, resulting in 10677 observations.

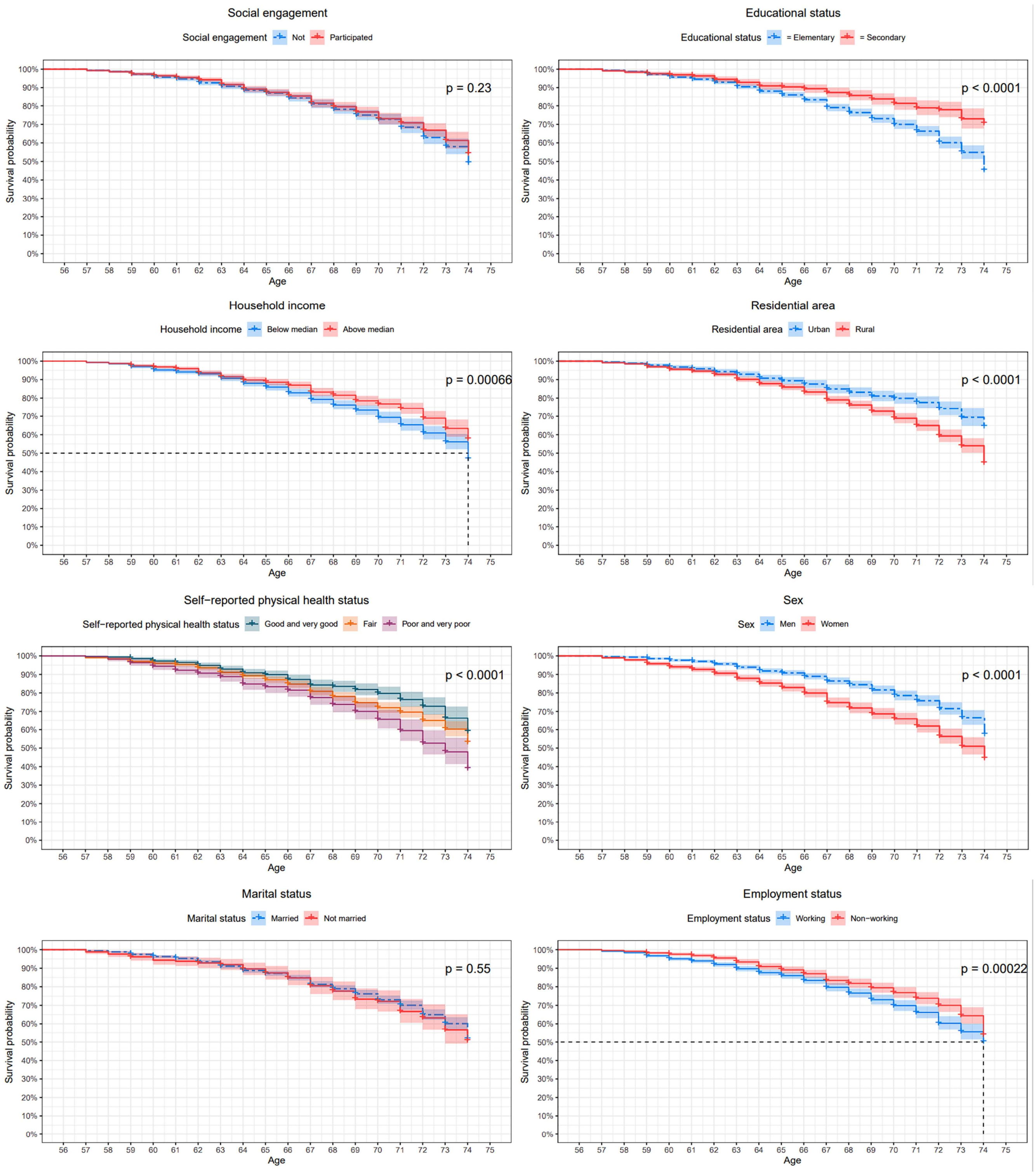

Figure. 7. Kaplan-Meier survival estimates of different groups for respondents aged 55-74.

Similar to the main analysis, the variable social engagement was treated as time-

fixed variables and only the measurement in Wave 1 was used, we then plotted Kaplan-Meier survival curves for area of residence, household income, marital status, employment status, and physical health status respectively.

The results of Kaplan-Meier survival analysis of different groups for respondents aged 55-74 (Figure 7) are consistent with those of the main analysis for respondents aged 55-77 (supplementary section F). In Figure 7, the first sub-plot showed that there was no significant difference between the two survival curves for those involved in social activities or not at the first measurement. The survival rate of the group with lower education (elementary education and below) was significantly lower than that of the group with higher education (secondary education and above). The survival rate of the group with lower household income was significantly lower than that of the group with higher household income. The group living in the rural area had a higher risk rate of developing depression compared to the group living in the urban area, and this gap increased with age. Those who perceived themselves to be in poor health at the first measurement had a higher risk of depression, compared to those who perceived themselves to be in good health at the first measurement. Women had a significantly higher risk of depression than men. Marital status did not have a significantly different effect on the survival rate of depression. The non-working group had a higher survival rate than the non-working group.

Supplementary C.2 Bivariate survival analysis by sex

We conducted sex-specific Kaplan-Meier survival analysis to examine the sex differences of different risk groups.

As shown in Figure 8, the effect of educational status on developing depression was more significant among women than men. Both men and women with secondary education and above had a lower risk rate of depression compared to those with elementary education and below. For both men and women, those living in rural areas had a higher risk of depression compared to those living in urban areas. The effect of physical health status on depression was significant for both men and women. The groups who perceived themselves to be in good health had a lower risk of depression than those who perceived themselves to be in poor health. There was no significant difference for the two groups of different marital statuses in developing depression for both sexes. For both men and women, the working group had a higher risk of depression compared to the non-working group.

The results of sex specific Kaplan-Meier survival estimates of different groups for the age range of 55-77 (Figure 8) are consistent with the Kaplan-Meier survival estimates of different groups for age range of 55-77 (supplementary section F), except for social engagement and household income. There was no significant difference between the two survival curves for women, never engaged and engaged in social activities, whereas socially active men had a lower risk rate of depression compared to men who were not socially active. Differences in household income only influenced depression for men, but not for women. Men with the household income above the median had a lower risk rate of depression compared to men with the household income below the median.

Men play a dominant role in family, who are considered as household providers

especially among the older people in China. Wanklyn (49) stated that one will lose physical strength, control, independence, and social power through the aging process, which contradicts with the image of men in social norms. Less social support significantly correlates with psychological distress among men.(50) Results of survival analysis above show that social engagement and household income are stronger protectors for depression among the older people in men than women.

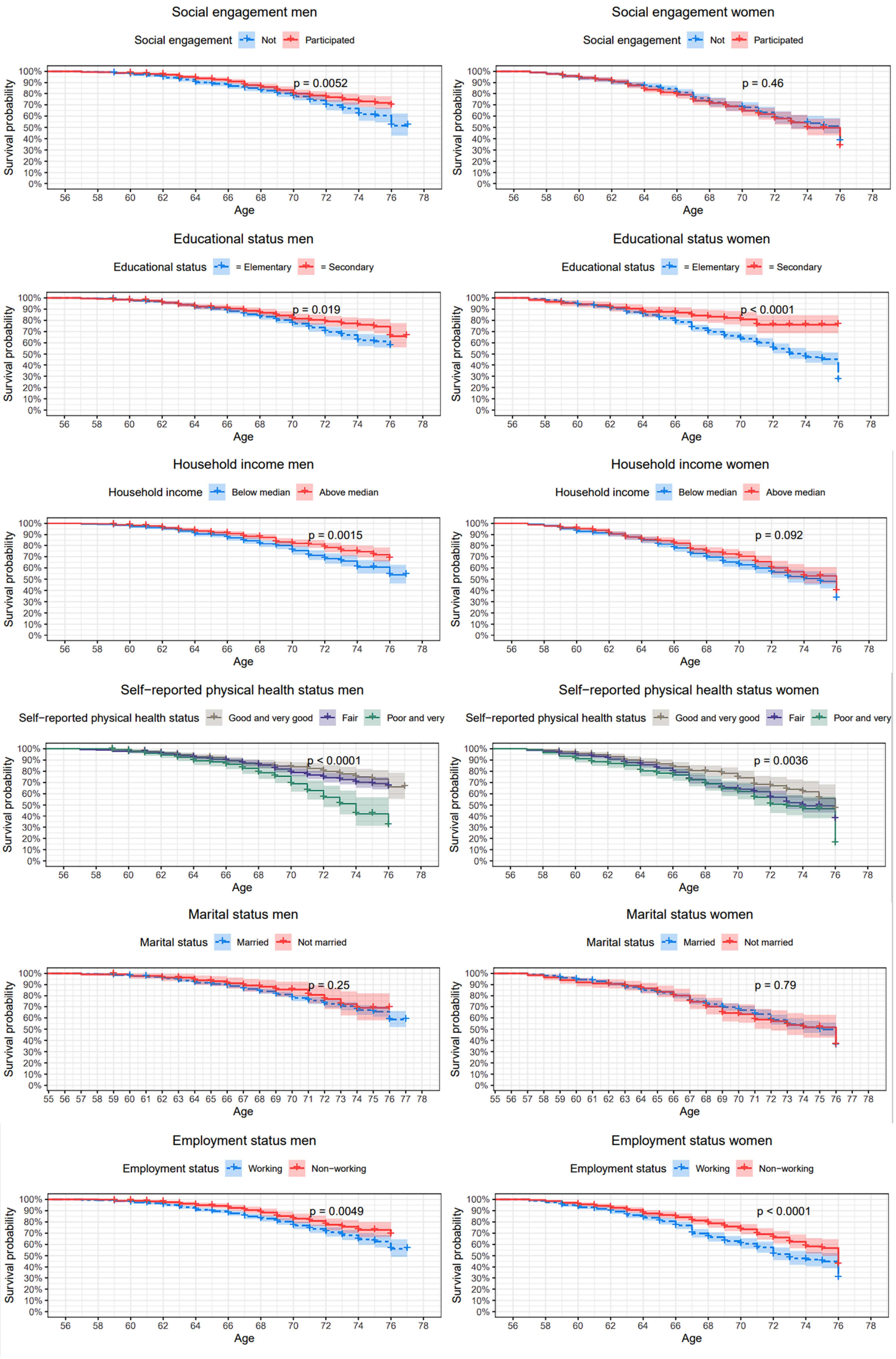

Figure. 8. Sex specific Kaplan-Meier survival estimates of different groups for respondents aged 55-77.

Supplementary C.3 Quadratic and cubic fit of age in general linear model.

To further examine in the relationship between social engagement and depression in older adults, we fitted a binomial generalized linear model with log-log link function, including age, the quadratic and cubic terms of age, sex, social engagement, educational status, marital status, urban-rural residential area, employment status, self-reported physical health status, and household income.

Results are shown in Table 4 model 1. The factors that had a significant effect on depression were social engagement, household income, physical health status, marital status, sex, and urban/rural residential area.

Table 4 Estimates of the effect of different variables on depression among respondents aged 55-77, controlling age, sex, educational status, annual household income, residential area, self-reported physical health status, marital status, and employment status.

		**Model 1**		**Model 2**		**Model 3**		**Model 4**		**Model 5**	
		**Hazard ratio**	**95% Confidence Interval**	**Hazard ratio**	**95% Confidence Interval**	**Hazard ratio**	**95% Confidence Interval**	**Hazard ratio**	**95% Confidence Interval**	**Hazard ratio**	**95% Confidence Interval**
**Social engagement (Reference: Not participated)**											
	Participated	0.881*	[0.788,0.985]	0.891*	[0.797,0.995]						
**Interpersonal activities (Reference: Not participated)**											
	Participated					0.856	[0.570,1.285]	0.890*	[0.795,0.994]		
**Voluntary activities (Reference: Not participated)**											
	Participated					0.944	[0.704,1.267]	0.980	[0.779,1.239]		
**Learning activities ((Reference: Not participated))**											
	Participated					1.146	[0.681,1.929]	1.110	[0.668,1.837]		
**Frequency of social activities (Reference: Never)**											
	Not regularly					1.100	[0.723,1.674]				
	Almost every week					1.113	[0.706,1.755]				
	Almost daily					0.985	[0.637,1.524]				
**Frequency of Interpersonal activities (Reference: Never)**											
	Not regularly									0.929	[0.781,1.105]
	Almost every week									0.946	[0.785,1.141]
	Almost daily									0.845*	[0.734,0.973]
**Frequency of Voluntary activities (Reference: Never)**											
	Not regularly									1.097	[0.851,1.414]
	Almost every week									0.720	[0.357,1.452]
	Almost daily									0.573	[0.239,1.377]

**Frequency of Learning activities**											
**(Reference: Never)**											
	Not regularly									1.177	[0.166,8.336]
	Almost every week									0.819	[0.206,3.264]
	Almost daily									1.195	[0.684,2.088]
**Sex (Reference: Men)**											
	Women	1.413***	[1.261,1.585]	1.403***	[1.251,1.573]	1.410***	[1.257,1.581]	1.400***	[1.252,1.574]	1.412***	[1.259,1.583]
**Educational status (Reference: ≤**											
**Elementary)**											
	≥ Secondary	0.887	[0.768,1.025]	0.867	[0.751,1.001]	0.862*	[0.746,0.996]	0.870*	[0.748,0.999]	0.869	[0.751,1.004]
**Marital status (Reference: Married)**											
	Not married	1.334***	[1.156,1.539]	1.317***	[1.142,1.520]	1.315***	[1.140,1.518]	1.320***	[1.141,1.520]	1.318***	[1.142,1.521]
**Employment status (Reference:**											
**Working)**											
	Non-working	0.900	[0.794,1.020]	0.899	[0.794,1.019]	0.904	[0.797,1.025]	0.900	[0.792,1.018]	0.906	[0.799,1.027]
**Household income (Reference: Below**											
**the median)**											
	Above the median	0.833**	[0.740,0.938]	0.834**	[0.741,0.939]	0.835**	[0.742,0.940]	0.830**	[0.741,0.939]	0.835**	[0.742,0.940]
**Physical health status (Reference:**											
**Good and very good)**											
	Fair	1.785***	[1.521,2.096]	1.791***	[1.526,2.103]	1.788***	[1.523,2.009]	1.790***	[1.527,2.103]	1.790***	[1.525,2.101]
	Poor and very poor	3.567***	[3.000,4.241]	3.561***	[2.995,4.234]	3.553***	[2.987,4.225]	3.560***	[2.995,4.236]	3.554***	[2.988,4.226]
**Residential area (Reference: Urban)**											
	Rural	1.238***	[1.085,1.413]	1.235**	[1.083,1.409]	1.235**	[1.081,1.409]	1.240**	[1.085,1.413]	1.233**	[1.080,1.407]
**Age**		12052.265***	[96.667,1502652.494]	1.032***	[1.020,1.044]	1.032***	[1.020,1.044]	1.030***	[1.020,1.044]	1.032***	[1.020,1.044]
**Age^2**		0.870***	[0.808,0.937]								
**Age^3**		1.001***	[1.000,1.001]								

Model 1: Estimates of the effect of social engagement on depression. Model 2: Estimates of the effect of social engagement on depression. Model 3: Estimates of the effect of types of social activities and frequency of engaging social activities on depression. Model 4: Estimates of the effect of types of social activities on depression. Model 5: Estimates of the effect of frequency of engaging social activities (matching with the 3 types of social activities) on depression. Note: Respondents=2891, observations=10,854.

Supplementary C.4 Using age as continuous variable

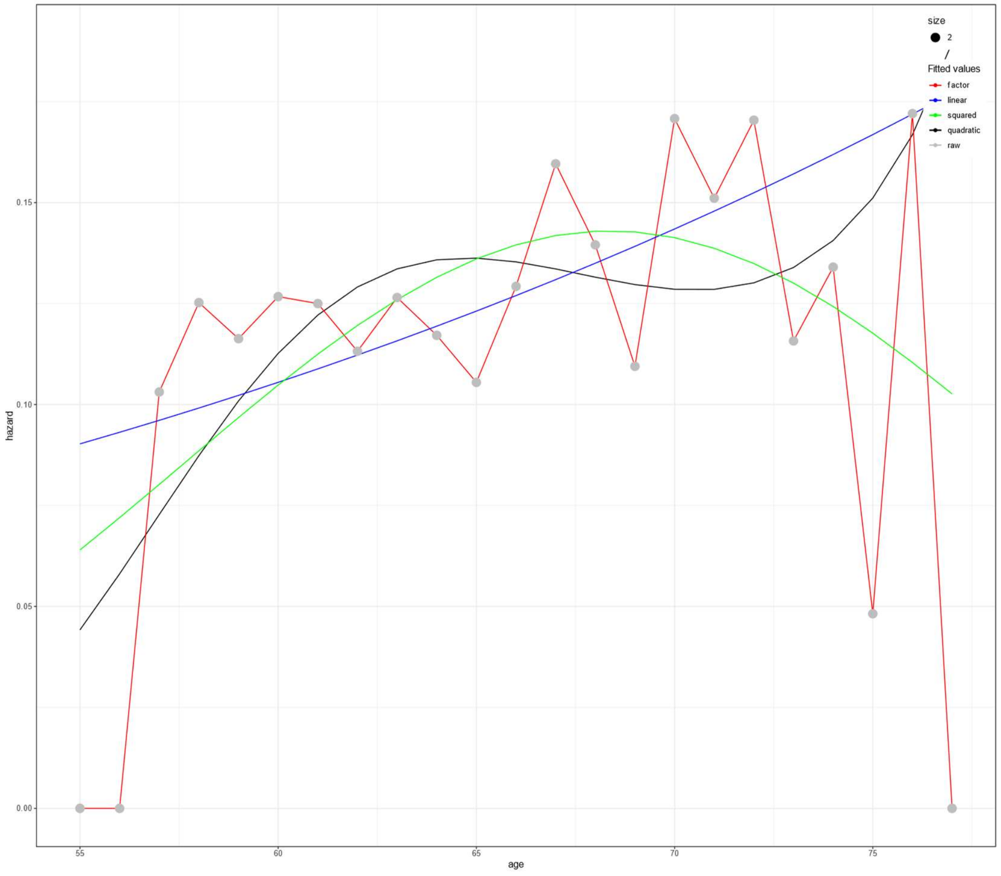

Figure. 9. Age as continuous variable is linear with depression.

Figure 9 shows that using age as a continuous variable is linear with the outcome variable depression.

Supplementary Section D. Effect of Social Engagement on Depression among Chinese Older People

Model 2 in Table 4 shows the association between social engagement and depression in older adults after controlling for confounders (sex, educational status, annual household income, residential area, self-reported physical health status, marital and employment status). This shows that the group that had been socially engaged at the first wave were 11% less likely to be depressed in later waves than the group that had not been socially engaged [HR 0.89, 95% CI 0.80 to 0.99], all else equal.

We added 2 more new variables: (1) types of social activities: interpersonal activities, voluntary activities and learning activities; (2) frequency of engaging these social activities. All were obtained from Wave 1.

Since the original questionnaire had multiple choices of types of social activities, the participation in each category of activity was treated as a separate variable, with 1 indicating participation in such activities and 0 indicating no participation[1].

Adding the 2 variables above to the model would cause high correlation and none of the 2 variables of interest would have significant coefficients, as shown in model 3 of Table 4. The 2 variables have high correlation between themselves, the coefficients

[1] The number of participation activities is selected in the original questionnaire from CHARLS DA056 except for the items 'other' and 'did not participate in any of the above'. DA057 from the 2011 questionnaire recorded the frequency of participation based on the activities selected in DA056 (options 1 to 10), we selected the maximum of these participation frequencies as the frequency of social activities variable. Item 'other' was categorized as not engaging in social activities. 23 respondents chose 'other' in DA056, and there's no corresponding frequency towards 'other'. One respondent had missing value of frequency. For all of these 24 respondents, I set its participation frequency to 0 i.e.: never participate in activities.

for interpersonal activities and the frequency of engaging almost daily is 0.58.

In the model 4 shown in Table 4 which only included types of social activities rather than all 2 variables above, we found that out of the three types of social activities, respondents engaging in interpersonal activities were 11% less likely to develop depression than those not engaging in interpersonal activities.

When adding types of social activities and their corresponding frequencies of engagement in the model as shown in model 5 of Table 4, only the frequency of almost daily involvement in interpersonal activities is significantly associated with depression with a hazard ratio of around 0.85, indicating that the group engaged in interpersonal activities almost daily in Wave 1 were 15% less likely to suffer from depression.

Supplementary Section E. Baseline descriptive statistics

Table 5 Summary of the social, economic, and demographic characteristics of the baseline sample with CESD scores.

Respondents=9141	**Wave1 (Percentage)**		**Wave2 (Percentage)**		**Wave3 (Percentage)**		**Wave4 (Percentage)**	
Observations=36564	9141		9141		9141		9141	
**Age groups**								
<45	306	(3.4)	138	(1.5)	67	(0.7)	21	(0.2)
45-54	3228	(35.3)	2665	(29.2)	2219	(24.3)	1303	(14.3)
55-64	3742	(41.0)	3873	(42.4)	3700	(40.5)	3473	(38.0)
65-74	1572	(17.2)	1993	(21.8)	2463	(27.0)	3126	(34.2)
75-84	278	(3.0)	451	(4.9)	655	(7.2)	1097	(12.0)
≥85	6	(0.1)	12	(0.1)	28	(0.3)	112	(1.2)
**Sex**								
Men	4162	(45.5%)	4162	(45.5%)	4162	(45.5%)	4162	(45.5%)
Women	4979	(54.5%)	4979	(54.5%)	4979	(54.5%)	4979	(54.5%)
**Marital status**								
Married	7872	(86.1)	7755	(84.8)	7606	(83.2)	7269	(79.5)
Not married	1269	(13.9)	1386	(15.2)	1535	(16.8)	1872	(20.5)
**Educational status**								
≤ Elementary	6216	(68.0)	6216	(68.0)	6216	(68.0)	6216	(68.0)
≥ Secondary	2925	(32.0)	2925	(32.0)	2925	(32.0)	2925	(32.0)
**Residential area**								
Urban	3092	(33.8)	3092	(33.8)	3092	(33.8)	3092	(33.8)
Rural	6049	(66.2)	6049	(66.2)	6049	(66.2)	6049	(66.2)
**Physical health status**								
Good and very good	2094	(22.9)	2021	(22.1)	1974	(21.6)	1955	(21.4)
Fair	4598	(50.3)	4943	(54.1)	4992	(54.6)	4435	(48.5)
Poor and very poor	2449	(26.8)	2177	(23.8)	2173	(23.8)	2746	(30.1)
**Total household income**								
Below the median	4837	(52.9)	4837	(52.9)	4837	(52.9)	4837	(52.9)
Above the median	4304	(47.1)	4304	(47.1)	4304	(47.1)	4304	(47.1)
**Employment status**								
Working	6373	(69.7)	6482	(70.9)	6106	(66.8)	5731	(62.7)
Non-working	2768	(30.3)	2659	(29.1)	3035	(33.2)	3409	(37.3)
**Frequency of attending social activities**								
Never	4551	(49.8)	4551	(49.8)	4551	(49.8)	4551	(49.8)
Not regularly	1365	(14.9)	1365	(14.9)	1365	(14.9)	1365	(14.9)
Almost every week	1059	(11.6)	1059	(11.6)	1059	(11.6)	1059	(11.6)
Almost-daily	2166	(23.7)	2166	(23.7)	2166	(23.7)	2166	(23.7)
**CESD score**								
0~9	5775	(63.2)	6155	(67.3)	5921	(64.8)	5506	(60.2)
10~30	3366	(36.8)	2986	(32.7)	3220	(35.2)	3635	(39.8)

Table 6 Summary of the social, economic, and demographic characteristics of the baseline sample without CESD scores.

Respondents=1071	**Wave1 (Percentage)**		**Wave2 (Percentage)**		**Wave3 (Percentage)**		**Wave4 (Percentage)**	
Observations=4284	1071		1071		1071		1071	
**Age groups**								
<45	34	(3.2)	25	(2.3)	15	(1.4)	7	(0.7)
45-54	247	(23.1)	203	(19.0)	179	(16.8)	103	(9.7)
55-64	330	(30.9)	327	(30.6)	291	(27.3)	279	(26.1)
65-74	270	(25.3)	269	(25.2)	287	(26.9)	298	(27.9)
75-84	160	(15.0)	202	(18.9)	228	(21.4)	263	(24.6)
≥85	26	(2.4)	41	(3.8)	67	(6.3)	117	(11.0)
**Sex**								
Men	480	(44.8)	480	(44.8)	480	(44.8)	480	(44.8)
Women	591	(55.2)	591	(55.2)	591	(55.2)	591	(55.2)
**Marital status**								
Married	879	(82.1)	832	(77.7)	799	(74.6)	733	(68.4)
Not married	192	(17.9)	239	(22.3)	272	(25.4)	338	(31.6)
**Educational status**								
≤ Elementary	841	(78.5)	841	(78.5)	841	(78.5)	841	(78.5)
≥ Secondary	230	(21.5)	230	(21.5)	230	(21.5)	230	(21.5)
**Residential area**								
Urban	365	(34.1)	365	(34.1)	365	(34.1)	365	(34.1)
Rural	706	(65.9)	706	(65.9)	706	(65.9)	706	(65.9)
**Physical health status**								
Good and very good	230	(21.5)	231	(21.6)	194	(23.7)	106	(20.4)
Fair	480	(44.8)	502	(46.9)	383	(46.7)	237	(45.6)
Poor and very poor	361	(33.7)	338	(31.6)	243	(29.6)	177	(34.0)
**Total household income**								
Below the median	583	(54.4)	583	(54.4)	583	(54.4)	583	(54.4)
Above the median	488	(45.6)	488	(45.6)	488	(45.6)	488	(45.6)
**Employment status**								
Working	563	(52.6)	543	(50.7)	508	(47.4)	436	(41.1)
Non-working	508	(47.4)	528	(49.3)	563	(52.6)	624	(58.9)
**Frequency of attending social activities**								
Never	614	(57.3)	614	(57.3)	614	(57.3)	614	(57.3)
Not regularly	117	(10.9)	117	(10.9)	117	(10.9)	117	(10.9)
Almost every week	98	(9.2)	98	(9.2)	98	(9.2)	98	(9.2)
Almost-daily	242	(22.6)	242	(22.6)	242	(22.6)	242	(22.6)

Based on the participants inclusion flowchart in Figure 1, out of the 11,988 respondents who actively engaged in all four waves of the study, 9,792 respondents had

CESD scores, while the remaining 2,196 respondents had missing CESD scores. After deleting observations with the missing CESD scores, the baseline descriptive statistics pertaining to the respondents with CESD scores in Table 5 and those with missing CESD scores in Table 6.

Supplementary Section F. Bivariate survival analysis for each of the confounders

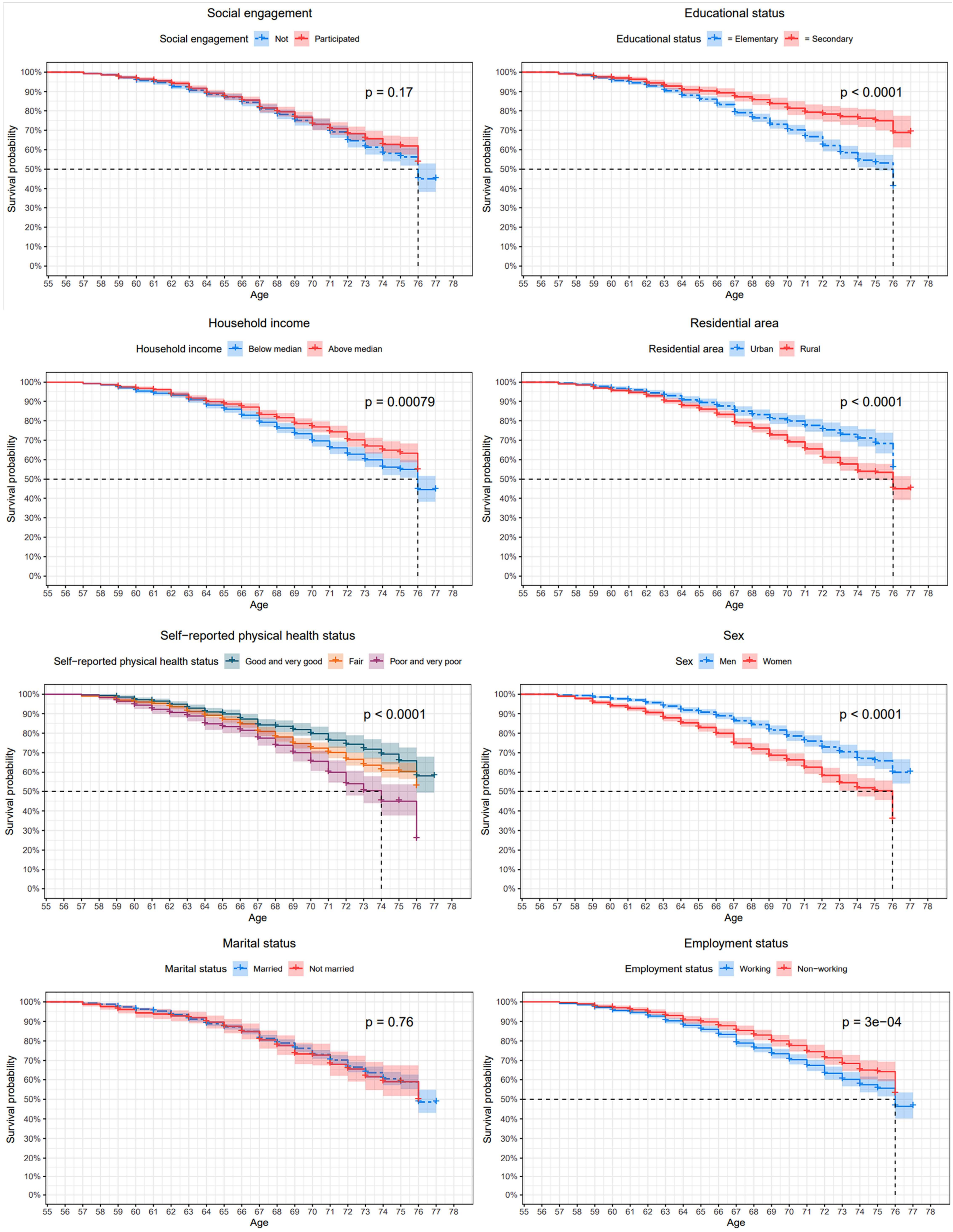

Figure. 10. Survival curves of different risk groups in developing depression for respondents aged 55-77.

Figure 10 shows the bivariate survival curves of different risk groups by 8 key

factors (social engagement, educational status, household income, residential area, sex, physical health status, employment status and marital status) respectively in developing depression for respondents aged 55-77. The survival rate here is defined as the probability of surviving, hence not developing depression. The first sub-plot shows that there was no significant difference ($p=0.170$) between the two survival curves with or without 2011's social engagement. The second sub-plot shows that the survival rate of the group with lower education (elementary education and below) was significantly lower ($p<0.001$) than that of the group with higher education (secondary education and above), whilst the gap between both groups widened with aging. In the third sub-plot, the curves show that respondents from higher income household had a significantly higher survival rate of depression than respondents with lower household income ($p<0.001$). The fourth sub-plot exhibits that the group living in the rural area had a significantly lower survival rate of depression compared to those living in the urban area ($p<0.001$), and this difference increased with age. As with self-reported health, the fifth sub-plot shows that there was a significant difference between the three curves with those with "good and very good" health as the reference group ($p<0.001$) and those with poor self-reported health at the lowest survival rate of depression. With the widening difference along age, women tended to have a significantly lower survival rate of depression than men in the sixth sub-plot ($p<0.001$). No difference was found for groups of different marital statuses in the seventh sub-plot ($p=0.760$). The last sub-plot shows that the group of non-working had a significantly higher survival rate of depression than those of working ($p<0.001$).

Supplementary Section G. Full model output of mediation analysis

Table 7 shows the respondents with secondary education and above were around 60% more likely to engage in social activities more frequently than those with elementary education and below [HR 1.60, 95% CI 1.48 to 1.74].

Table 7 Mediation analysis. Estimates of the average effect of educational status on social engagement frequency among respondents aged 55-77, controlling for age, sex, marital status, self-reported physical health status, and residential area.

		**Hazard ratio**	**95% Confidence Interval**	**P-value**
**Educational status (Reference: ≤ Elementary)**				
	≥ Secondary	1.606	[1.481,1.742]	<0.001
**Age**		1.007	[0.999,1.014]	0.074
**Sex (Reference: Men)**				
	Women	1.192	[1.106,1.284]	<0.001
**Marital status (Reference: Married)**				
	Not married	1.137	[1.026,1.261]	0.014
**Physical health status (Reference: Good and very good)**				
	Fair	0.982	[0.904,1.067]	0.670
	Poor and very poor	0.858	[0.768,0.958]	0.007
**Residential area (Reference: Urban)**				
	Rural	0.664	[0.615,0.716]	<0.001

References

47. Survey and Research Center for China Household Finance. Statista. [cited 2023 Mar 22]. China: 51-60 years old employment by gender 2011. Available from: https://www.statista.com/statistics/304632/china-51-60-years-old-employment-ratio-by-gender/

48. Survey and Research Center for China Household Finance. Statista. [cited 2023 Mar 22]. China: unemployment rate by age 2012. Available from: https://www.statista.com/statistics/304668/china-unemployment-rate-by-age/

49. Wanklyn P. Caring for Older People: Homes and Housing for Elderly People. BMJ. 1996 Jul 27;313(7051):218–21.

50. Wester SR, Christianson HF, Vogel DL, Wei M. Gender role conflict and psychological distress: The role of social support. Psychol Men Masculinity. 2007 Oct;8(4):215–24.
